# MicroRNAs in the Stressed Heart: Sorting the Signal from the Noise

**DOI:** 10.3390/cells3030778

**Published:** 2014-08-05

**Authors:** Scot J. Matkovich

**Affiliations:** Center for Pharmacogenomics, Department of Medicine, Washington University School of Medicine, St. Louis, MO 63130, USA; E-Mail: smatkovi@dom.wustl.edu; Tel.: +1-314-747-3455; Fax: +1-314-362-8844

**Keywords:** RNA-induced silencing complex, microRNA, ribosome, translation, hypertrophy, stress, heart, systems biology

## Abstract

The short noncoding RNAs, known as microRNAs, are of undisputed importance in cellular signaling during differentiation and development, and during adaptive and maladaptive responses of adult tissues, including those that comprise the heart. Cardiac microRNAs are regulated by hemodynamic overload resulting from exercise or hypertension, in the response of surviving myocardium to myocardial infarction, and in response to environmental or systemic disruptions to homeostasis, such as those arising from diabetes. A large body of work has explored microRNA responses in both physiological and pathological contexts but there is still much to learn about their integrated actions on individual mRNAs and signaling pathways. This review will highlight key studies of microRNA regulation in cardiac stress and suggest possible approaches for more precise identification of microRNA targets, with a view to exploiting the resulting data for therapeutic purposes.

## 1. Introduction

Cells need to respond to changes in their external environment to ensure continued optimum function and survival. This occurs not only through the actions of the proteins they initially manufacture in accordance with their tissue role, but by the capacity to alter their internal programming to turn the expression of groups of proteins on and off. Francis Crick’s “central dogma” of DNA-to-messenger RNA (mRNA)-to-protein focused attention on the activation or repression of mRNA transcriptional programs, which take place via mechanisms including altered promoter DNA methylation, histone-regulated DNA availability and transcription factor shuttling between the cytoplasm and nucleus. However, a large proportion of mammalian DNA is transcribed into noncoding RNA (which does not serve as mRNA). While noncoding RNAs can take many forms, investigations into the role of endogenous small, noncoding RNAs of approximately 22 nt in length (known as microRNAs) in suppressing the translation of mRNAs were sparked by Fire and Mello’s Nobel Prize-winning work on RNA interference by injected dsRNAs in *C. elegans* [1]. Like their mRNA cousins, the production of microRNAs is responsive to cues from the external environment and other cellular signals; microRNAs arise from introns spliced out of pre-mRNAs or via RNA polymerase II-driven transcription from their own promoters. Hairpin or ‘stem-loop’ pre-microRNAs are exported from the nucleus and then further processed into a duplex encoding two microRNA strands with different but partly complementary sequences. In most instances one strand (the guide) is primarily incorporated into RNA-induced silencing complexes (RISCs) while the other (passenger strand) is degraded, but in some cases, both the -5p and -3p strands of the duplex can be retained and inserted into RISCs, in a manner dependent on the thermodynamic stability of strand 5′ ends [2,3]. Thus, cellular reprogramming of protein translation involves not only alteration in coding mRNA levels, but also alterations in levels of noncoding microRNAs that serve to restrain expression of mRNAs. The study of microRNAs in cellular differentiation and organismal development, in stress-dependent signaling, and in diseases ranging from cancer to diabetes has delineated a constellation of individual microRNAs and target mRNAs involved in key cellular processes.

One reason that microRNAs have received much attention is the apparent capacity of individual microRNAs to regulate numerous downstream targets in related signaling pathways, and another is their relative ease of manipulation* in vivo* with reagents that can be considered as prodrugs. Traditional genetic overexpression or knockout studies of even single microRNAs have shown profound effects on cardiac gene expression, leading to amelioration or exacerbation of stress-induced cardiac phenotypes, and in some cases, disrupting mRNA translation in the heart sufficiently to cause spontaneous disease [4,5,6,7,8,9]. Several excellent, comprehensive reviews have been published recently that highlight roles for particular microRNAs in adaptive and maladaptive responses to demands for increased cardiac workload [10,11,12], in the response to myocardial infarction [13,14] and in the progression to heart failure [15,16,17], and it is not the primary intent of this article to re-tread the same ground. Rather, I would like to re-focus attention on several principles and practices that may guide interpretation of published studies and the planning of future investigations, but that are not always considered when integrating detailed mechanistic studies of specific microRNAs and their targets into a wider framework. The ability of microRNAs to engender large-scale changes in cellular behavior is the basis of both their potential and their peril, and suggests that a detailed understanding of which mRNAs they target, and how this may vary with context, is needed to fully understand the biology of these noncoding RNAs and thus to enable developing microRNA-based therapeutic strategies to be properly deployed. The experimental and analytic approaches outlined below may be valuable not just in studies and possible therapeutic uses of microRNAs in the heart, but also in a wide variety of microRNA-based investigations.

## 2. A ‘Systems’ Approach is Key to Understanding MicroRNA Signaling

An integrative or ‘systems’ approach is needed to understand the function of any gene or gene product in its appropriate cell or tissue context, and in the appropriate milieu of simultaneously occurring transcriptional and translational processes. Integrative approaches take into account the influence of individual biomolecules on related signaling processes, and current methods model biological pathways as so-called ‘scale-free’ networks, in which central ‘hub’ genes form vital links amongst relatively sparsely-connected entities [18,19]. In the case of microRNAs, the need for an integrative approach is made particularly acute by the predicted ability of microRNAs to influence a large number of downstream targets. As one example, studies from the Loscalzo and Chan laboratories using both informatic and experimental approaches have designated miR-21 as a critical ‘hub’ in multiple distinct processes leading to pulmonary hypertension [20]. However, there has been considerable difficulty in accurately and comprehensively defining mRNA targets of microRNAs* in vivo* (not only in the heart but in general). The relatively small degree of repression often observed for individual microRNAs on individual putative mRNA targets (consistent with a model of microRNA action that involves multiple co-operative effects on a large number of targets, rather than large effects on a few ‘dominant targets’, as suggested by findings from the ENCODE project [21] and others [22]) adds a further level of difficulty to the task. Other limiting factors likely relate to the fact that multiple, rather than single, microRNAs are regulated in response to stress with potentially co-operative or antagonistic actions, and that experimental validation of predicted microRNA targets is necessarily performed in cellular models that may not fully recapitulate the microRNA and mRNA environment of an intact heart responding to a stimulus. In addition, while even straightforward cataloging of microRNA targets to construct putative signaling networks needs to be completed, understanding which regulatory relationships between microRNAs and mRNAs may have the most powerful effects on the resulting phenotype will be critical to form useful, predictive models.

Thus, despite considerable progress in defining suppressive actions of microRNAs on particular mRNAs, there remain two major obstacles that must be overcome, regardless of tissue or disease context. The first is that of establishing which mRNAs a particular microRNA targets. Mammalian genomes encode ~1,000 microRNAs in comparison to ~20,000 mRNAs [23], and it is no small challenge to predict *a priori* which mRNAs may be subject to microRNA action. Amongst many variables, the mammalian requirement for only partial sequence complementarity, and the differing accessibility of potential microRNA binding sites arising from the influence of mRNA secondary structures, are two of the most important that complicate prediction of likely microRNA-mRNA interactions. Almost any review or experimental article on microRNA biology will bemoan the long list of probable false positives and false negatives arising from even the most fastidious computational procedures, although it is worth noting that establishing lists of true positives and true negatives from current empirical data is almost as problematic. However, novel combined RNA cross-linking and informatic approaches established in recent years [24,25,26,27,28,29] are beginning to close the knowledge gap of which microRNA-mRNA interactions are biophysically possible. The second obstacle relates to the oft-repeated principle, based largely on sequence comparison but also indicated from RNA cross-linking studies, that an individual microRNA might regulate hundreds of mRNAs and that an individual mRNA may have many microRNA binding partners. Given this complexity, which microRNA alterations in response to stressors will meaningfully affect mRNA translation and which microRNA actions in a given state are dominant amongst the myriad that could occur? 

## 3. Assessing MicroRNAs Altered in the Stressed Heart

### 3.1. Which Altered MicroRNAs may be of the Most Biological Significance?

**Figure 1:**
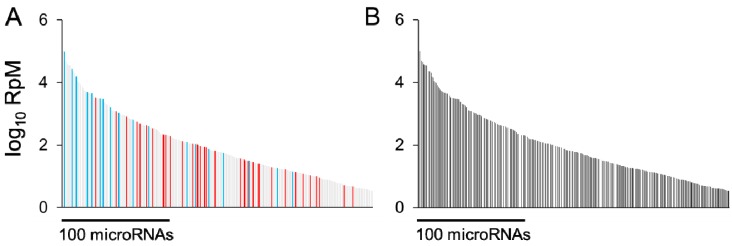
(**a**) 300 microRNAs expressed in mouse hearts, plotted according to abundance (log_10_ of Reads per Million microRNA reads, RpM), in descending order (light gray bars). Red bars denote microRNAs upregulated by at least 25%, false discovery rate (FDR) < 0.02, after 1 week pressure overload, as previously described [34]; blue bars denote microRNAs downregulated under the same conditions. (**b**) MicroRNAs stably expressed (not regulated) after 1 week pressure overload (*i.e.*, the light gray bars from (**a**)). For both (**a**) and (**b**), data are redrawn from [34] and NCBI GEO accession GSE56891. The most abundant 100 microRNAs represent 99% of the total cardiac microRNA content [35].

Having assayed microRNA expression in response to a physiological or pathological stimulus, an immediate question is which microRNA alterations underlie at least part of the observed phenotype. As an example, in the early response to cardiac pressure overload, a large number of microRNAs are regulated, spanning a wide range of abundances (Figure 1). One approach could be to choose a microRNA based on any or all of its degree of regulation, its abundance, and its possible targets, followed by replication of its regulation in a cell model or genetically engineered animal, and assess whether this is sufficient to provoke a spontaneous phenotype, e.g., [6,7,30,31,32,33]. In a genetically engineered animal, the same stimulus could be applied with exacerbated or reversed regulation of the microRNA of interest and the effect on phenotype studied. In the absence of a suitable genetic model, viral microRNA overexpression or anti-microRNA administration may be possible.

Of course, there are many limitations inherent in an approach of altering just one signaling molecule and measuring only a broad phenotypic readout, in an attempt to find the one or the few most important to a given response. Chief among these is the assumption that changes in phenotype are the result of independent signals and that synergistic actions of several co-regulated entities are minor contributors. No less important is the demand on resources needed to evaluate enhancement or inhibition of ~60 different microRNAs* in vivo* (in the example of early pressure overload shown in Figure 1). In this respect the study of microRNAs is no different to the study of any other biomolecule. We could employ brute-force, one-at-a-time overexpression and knockdown strategies to try to find a single approach that best ameliorates a disease, but we could also develop an understanding of how each component works together with others to influence biological responses. In the case of microRNAs, it may not necessarily be the case that the most abundant or the most strongly regulated microRNA(s) are the most important microRNA contributors to pathological responses to cardiac stress; it depends which mRNAs they suppress and to what extent. While relatively uncommon, mature microRNAs do exhibit genomic sequence variation with downstream signaling consequences, as typified for miR-96 [36] and miR-499 [37]. An additional source of variation arises from 5′-isomers (isomiRs) that cause a frameshift in the ‘seed’ sequence of a given microRNA. In the case of the archetypal cardiac microRNA miR-133a, two 5′-isomiRs are present in almost equal proportions and the mRNAs targeted by each of these isomiRs may differ [38]. Taken together, these variables beg the question of how best to determine which mRNAs are targeted by regulated microRNAs.

### 3.2. Challenges Faced by MicroRNA Target Prediction Algorithms

As noted in Section 2, identifying which mRNAs are subject to microRNA action* in vivo* is a challenge. No matter the computational approach taken, predicting such interactions in the absence of experimental data, especially quantitative data that can define whether individual microRNAs or mRNAs are indeed present in the cell or tissue of interest, forces any algorithm between a rock and a hard place. Without the ability to narrow the search space to the few hundred microRNAs and ~10,000 mRNAs reliably detected in the heart, and to rank the degree to which predicted interactions are likely to occur on the basis of stoichiometry, a large trade-off needs to be made between prediction sensitivity and specificity. Algorithms for microRNA target prediction employ to varying degrees parameters such as cross-species conservation of putative binding sites (since sequence conservation implies a relationship to function), free energy calculations for microRNA-mRNA hybrids, local GC content, and the degree to which hybridization of microRNA nucleotide 2–7 ‘seed’ sequence occurs. Some examples amongst many web-accessible, user-friendly engines are TargetScan [39,40], Pictar [41], and miRDB [42,43]. However, as new experimental data are reported, such as the binding of microRNAs to noncanonical target sites [44], the observation that microRNAs often appear to bind outside the originally-defined 3′ UTR of mRNAs [45], and the influence of microRNA sequence in regions downstream of the ‘seed’ [37,40,46] there appears to be a constant need to update the ‘rules’ under which these predictors operate. A lateral approach to overcoming prediction difficulties assumes that microRNAs target mRNAs in biologically-related signaling pathways, and assesses whether predefined functional gene sets are enriched in binding sites for a given microRNA (mirBridge; [47]). Nonetheless, such enrichment procedures do themselves depend on the veracity of the direct microRNA-mRNA interactions used as input.

Until recently, statements from various sources suggesting that a large number of direct microRNA-mRNA interactions involving the same microRNA could take place within cells seemed to be at odds with the numbers that had been experimentally validated. The advent of CLIP (cross-linking and immunoprecipitation) techniques (discussed further in Section 3.3.1) has revealed a wide variety and large number of possible microRNA-mRNA interactions, and integration of these data sets with other sequence- and structure-based prediction engines [27] has permitted a dialog between computational prediction and experimental observation to begin [28,29]. Nonetheless, we are still some distance from integrating parameters derived from whole transcriptome-based prediction algorithms together with empirical data from hearts, not to mention other tissues, beyond model cell systems. As a consequence, the most appropriate methods for identifying microRNA-dependent, differentially regulated mRNAs in the context of cardiac stress would still appear to be those that are primarily grounded in experimental measurements of microRNA and mRNA abundance and interactions, with informatic procedures serving to assist rather than to drive the analysis.

### 3.3. Quantifying MicroRNA-Dependent Regulation of mRNAs

**Figure 2:**
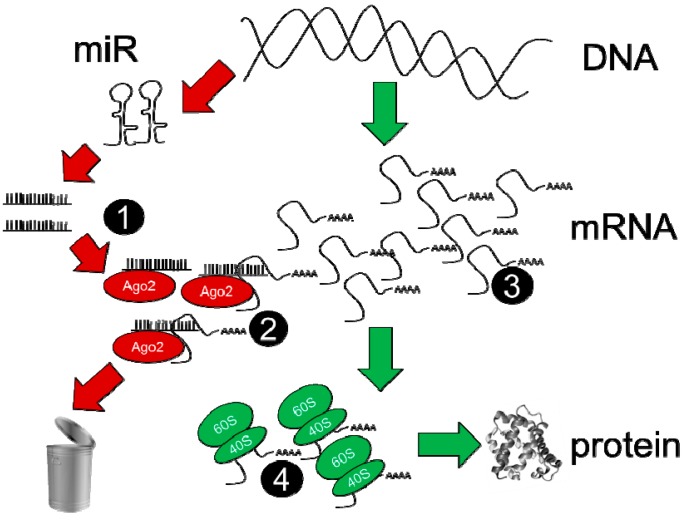
Quantitative and comprehensive ‘next-generation’ sequencing determinations used to measure mature microRNAs (step 1), to measure RISC-bound mRNA at step 2, to measure global RNA at step 3 and to measure ribosome-translated mRNA at step 4.

There are at least four steps of the microRNA-mRNA regulatory process that need to be assayed in order to determine which mRNAs are regulated by individual microRNAs, and to what degree; these are described schematically in Figure 2. Assessment of overall microRNA and mRNA expression in steps 1 and 3 is relatively straightforward and may be accomplished by either microarray or RNA-sequencing techniques. While microarrays represent a more mature technology platform for which most of the problematic data analysis issues have likely been worked out, it is difficult to dispute that RNA-sequencing offers a more quantitative platform for defining abundances of RNAs (enabling stoichiometric calculations to be attempted), enables easier detection of spurious signals (via knowledge of the exact sequence of RNA fragments) and has a theoretically limitless dynamic range [48]. Importantly, overall microRNA expression level (assessed by whole-cell assays) correlates well with microRNA abundance in the RISC [34]. Steps 2 and 4 represent key junctures for the evaluation of microRNA-dependent mRNA regulation; data from these assays reveal whether the binding of a particular mRNA is increased in the RISC, indicating greater targeting by microRNAs (step 2), while quantitation of ribosomal mRNA binding reflects the degree to which translation has been altered (as an alternative to quantitative proteomic methods). Numerous variations are possible in these assay steps, and merit separate discussion below in Section 3.3.1, Section 3.3.2 and Section 3.3.3.

#### 3.3.1. Assays for mRNA Presence in, and Targeting by, the RISC

**Figure 3:**
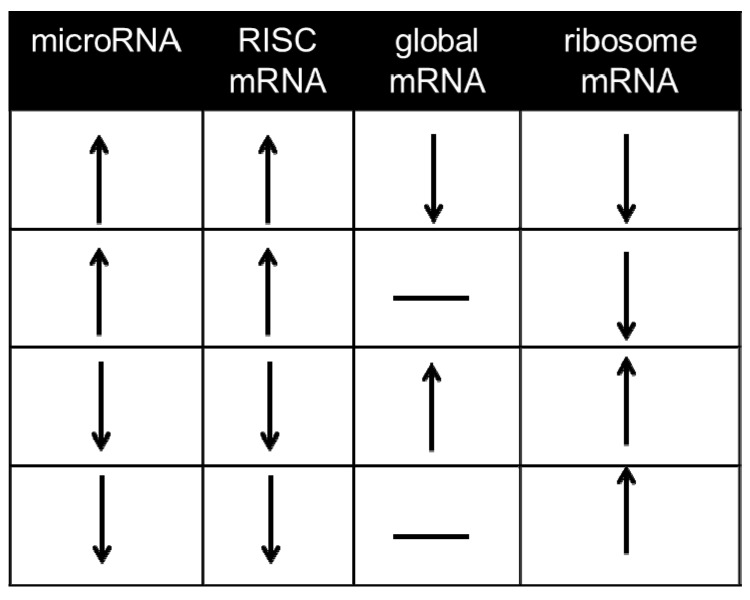
Regulatory parameters used to define microRNA-dependent mRNAs. Since suppression of mRNA translation without detectable decreases in global mRNA content has been widely reported in mammalian cells [45,60,61], it is worth considering as microRNA-dependent mRNAs those mRNAs whose abundance changes in the RISC in response to a given stimulus without exhibiting changes of global mRNA abundance (rows 2 and 4). mRNAs that do not exhibit alterations consistent with any one of these four rows are not considered to be microRNA-dependent under the paradigm outlined in this article.

mRNAs undergoing suppression in the RISC can be captured via immunoprecipitation of the microRNA-binding Ago protein, either with isoform-specific antibodies or pan-Ago antibodies, or alternatively via pulldown of the Ago binding partner and RISC scaffold GW182 [49,50,51]. Most mammalian studies to date have used immunoprecipitation of endogenous Ago2, e.g., [7,34,52,53,54,55,56,57]; some* in vitro* studies have employed an epitope-tagged Ago2 in cultured cells to facilitate retrieval of RISC-bound mRNA [51,58,59]. As pointed out by Karginov* et al.* [58] an important consideration when performing measurements of differential mRNA-binding in the RISC is to determine, in parallel, whether the initiating stimulus altered global levels of the same mRNA in the same direction,* i.e.*, through alterations in primary transcription, and whether the changes in the RISC simply reflect a greater abundance of the same mRNAs in the cell. Our laboratory does not classify such mRNA alterations as primarily microRNA-dependent (Figure 3). Assays of this kind are able to define whether or not the microRNA alterations arising as part of a stress response have caused sufficient reprogramming of the RISC to change the degree to which an individual mRNA is associated with the RISC, which on its own often comprises valuable data [34]. However, they do not offer empirical data on direct, individual microRNA-mRNA interactions and interaction data from hearts overexpressing or without microRNAs of interest are not a full substitute [7,37,55]. To this end, a number of crosslinking approaches have been developed.

The first of these, HITS-CLIP (high throughput sequencing with crosslinking and immunoprecipitation), employs UV irradiation to generate covalent crosslinks between protein and a directly bound RNA species [52]. Subsequent Ago purification and size-selection enables Ago:microRNA and Ago:microRNA:mRNA complexes to be distinguished, and the RNA fragments protected by Ago binding are isolated and quantitated. The data obtained reveal microRNAs and mRNAs present in the RISC, but pinpointing exact microRNA interaction sites on mRNAs, thus revealing the identities of microRNAs that regulate individual mRNAs, was not initially described as possible (although see below for recent refinements to the technique). Reconstitution of likely binding maps relied upon fitting the most likely microRNA (obtained from hybridization rules defined *a priori*) to a given Ago-protected mRNA fragment. Notably, HITS-CLIP can be performed both from cultured cells and from intact tissue such as brain slices [52], although no such studies have yet been reported from intact hearts. An extension of HITS-CLIP known as CLASH (crosslinking, ligation and sequencing of hybrids) exploits the fact that microRNAs and mRNAs present on the same Ago molecule are able to be ligated together, following crosslinking and partial hydrolysis [59]. As a result, so-called ‘chimeric’ reads are obtained via RNA-sequencing, in which the 5′ segment corresponds to the microRNA of interest and the 3′ segment of the same read corresponds to its mRNA binding site. While CLASH was initially described in HEK293 cells overexpressing epitope-tagged Ago, the presence of chimeric reads corresponding to microRNA-mRNA ligation has been reported from human pancreatic islet cells subjected to Ago2 HITS-CLIP [62], suggesting that this approach will also be useful* in vivo*.

Another technique, PAR-CLIP, takes advantage of a specific ‘lesion’ created by the UV crosslinking of nucleic acid analogues such as 4-thiouridine (4SU) to protein when RNA subsequently undergoes reverse transcription. Prior cell labeling and incorporation of 4SU into RNA leads to T-to-C transitions in resulting cDNA, meaning that the precise site of mRNA interaction with Ago2 can be revealed via identifying mRNA fragments containing specific T-to-C ‘mismatches’ [51]. Refinements to the original method have been published to increase specificity [25] and other groups have enhanced the bioinformatic procedures necessary to distinguish specific 4SU-induced ‘lesions’ from other sequencing errors or polymorphisms [26]. Like noncrosslinking RISC immunoprecipitation and the originally-described HITS-CLIP method, PAR-CLIP is unable to distinguish exact sites of microRNA interaction and the identities of interacting microRNAs, but the search space for interacting microRNAs is limited to the nucleotides immediately surrounding the T-to-C ‘lesion’. Interestingly, improved HITS-CLIP data analysis methods have observed an increased frequency of reverse transcriptase errors at the nucleotides involved in crosslinking to RNA-binding proteins (CIMS, or cross-link-induced mutation sites) [24]. While these errors are not as prevalent as in e.g., RNA labeled to equilibrium with 4SU and their variety adds additional challenges when analyzing RNA-sequencing data, their occurrence suggests that proper analysis of HITS-CLIP data may offer some of the benefits of PAR-CLIP methods without the need to prelabel cells of interest (which is an obvious barrier to application of PAR-CLIP in intact tissue). Furthermore, recent studies have identified nucleolar stress and rRNA depletion induced by 4SU labeling, which may represent a confounder in the interpretation of PAR-CLIP experiments [63].

Data obtained on individual microRNA-mRNA interactions from cardiomyocyte-like cell lines may not correspond well to the interactions taking place within cardiomyocytes of the intact heart, even less within cardiomyocytes of the stressed heart (see Section 5). This places a limit on the applicability of data derived from CLIP techniques in cultured cells. An ingenious possible solution to this issue involves addition of anti-miRs against a microRNA of interest to lysates generated for ‘standard’, noncrosslinking RISC immunoprecipitation procedures; the anti-miR competes with bound mRNAs for access to Ago2 and mRNA levels in the immunoprecipitate can be measured with and without anti-miR present [64]. While this has been validated only for liver lysates and miR-122 at present [64], it is likely that a similar approach can be applied to a wide variety of tissues.

There is a clear need to determine the overlap in microRNA-mRNA regulatory information gained from these techniques, in a cell or tissue system at baseline, and when that same cell or tissue system is subjected to an identical stimulus (and thus the same reprogramming of the RISC) prior to analysis by multiple techniques. HITS-CLIP, PAR-CLIP and CLASH provide largely overlapping data sets in HEK293 cells cultured under basal conditions [59,65] but whether they exhibit the same overlap in regulatory data when performing stimulus *vs.* control analyses is not known. The correspondence of differential RISC mRNA abundance and microRNA-binding data obtained from CLIP techniques with the RISC mRNA abundance data (and inferential microRNA-mRNA binding data) obtained from noncrosslinking approaches has also not been established. The establishment of a CLIP method in the intact heart and its comparison to existing RISC immunoprecipitation data sets would be highly valuable for directing the best approach to use in future experiments aimed at deciphering direct microRNA-mRNA interactions in the heart and their changes in response to stimuli.

#### 3.3.2. Pinning the MicroRNA to the Target—Approaches in the Absence of CLIP Experiments

Two previous studies from our laboratory have predicted changes in individual cardiac microRNA-mRNA interactions, using as input differentially expressed microRNAs and mRNAs [35] or differentially expressed microRNAs and microRNA-dependent mRNAs, defined as shown in Figure 3 using noncrosslinking RISC immunoprecipitation and requiring the more stringent criteria of opposing regulation in RISC and global mRNA [34]. The relatively small number of altered microRNAs and altered mRNAs defined in these studies facilitated the use of a simplistic, sequence-based comparison, which if applied to the entire known microRNA and mRNA transcriptomes would result in extremely poor specificity but had the advantage, in this context, of offering high sensitivity by only limited enforcement of the RNA interaction ‘rules’ used by other algorithms [39,40,41,43]. While helpful in obtaining putative microRNA-mRNA pairs for further validation, this approach did not attempt to take into account the importance of mRNA secondary structure in the vicinity of microRNA-binding sites (modeled by e.g., Mfold [66] or Sfold [67]), the free energy of sterically unrestricted binding, calculated by e.g., the RNAhybrid tool [68], the possible requirement for ordered secondary structure in the 5′UTR (not the 3′UTR) of microRNA-targeted mRNAs [69] and steric constraints on microRNA conformation imposed by the Ago binding pocket [27]. A number of these structural considerations have been allied to HITS-CLIP and PAR-CLIP data sets by the Sfold-STarMir web server, which has recently become publicly available [29]. This suite of procedures integrates linear sequence considerations, RNA secondary structure estimations and empirical data to make its binding predictions. With the inclusion of a machine learning approach, STarMir is capable of inferring microRNA-mRNA binding likelihood even for RNAs that are not represented in the underlying CLIP training data sets [28]. The continued development of this and other tools should be highly valuable in the interpretation of microRNA and mRNA changes in experimental situations where CLIP techniques prove difficult to establish.

In our workflow, RISC and ribosome profiling (see Section 3.3.3.) experimental data always serve as the primary criteria for classifying mRNAs as regulated by microRNAs* in vivo*; the informatic procedures only assist in attributing the regulation of particular mRNAs to the action of particular microRNAs. A key advantage of next-generation sequencing procedures is their ability to accurately quantitate RNA abundances, and thus to allow consideration of microRNA and mRNA stoichiometry when deciding which among several potential microRNA binding partners may be the most important contributors to downregulating a particular mRNA. There are two further, important considerations for appropriate data interpretation, which are applicable to both crosslinked and non-crosslinked immunoprecipitation methods: (1) assays using material from whole hearts naturally include RNA from cardiomyocytes and non-cardiomyocytes. While some RNAs are highly enriched in one cell fraction *vs* the other, many are more evenly distributed, and assessing whether these are altered in one or both fractions will require cell separation prior to RNA isolation. Preliminary determinations of RNA abundances in each cell fraction, prior to experiments examining effects of cardiac stress, should be valuable; (2) while RNA-sequencing procedures are highly quantitative in comparison to array methods or RT-qPCR, when evaluating RISC RNA assays it is only really possible to quantitate any RISC RNA *vs.* any other RISC RNA. Without determining what proportion of total cellular RNAs are bound to Ago proteins and captured by RISC immunoprecipitation, the measured abundances of RNAs in the RISC cannot be directly related to the amount of cellular mRNA that is undergoing RISC-mediated suppression. Nonetheless, alterations in RISC mRNA abundance should be indicative of changes at the whole-cell level.

#### 3.3.3. Assays for Altered Translation of mRNAs

In a similar manner to the direct and indirect data on microRNA-mRNA interactions captured by RISC RNA assessment *vs* measurement of global mRNA, the ultimate effects on mRNA translation can be captured by a direct method (mRNA ribosome profiling) or less direct methods (proteomic or single-protein evaluations). Neither approach gives information on the responsible microRNAs; this is the task of the previously mentioned assays (Figure 2, steps 1–3); but they serve to demonstrate that the previously acquired data can be directly related to altered translational output, rather than being used merely to infer the outcome. Ribosome profiling methods employ cell lysis in the presence of cycloheximide (to arrest ribosome translation) followed by RNase-mediated ‘trimming’ of RNAs not protected by ribosome binding [70], and can be applied to flash-frozen solid tissue [71]. Subsequent procedures recover ribosomes on the basis of buoyant density, isolate RNAs, perform reverse transcription, deplete rRNA content and prepare remaining cDNAs for high-throughput sequencing [70]. While technically demanding, ribosome profiling assays have given unprecedented insight into the mechanisms by which microRNAs may inhibit translation [61,69] and should be highly valuable for confirming microRNA-dependent suppression for those mRNAs observed to be altered in their RISC abundance (Figure 2, step 2), but not necessarily at the global mRNA level (Figure 2, step 3).

Evaluation of the protein products of mRNAs is the more common approach for validating microRNA effects on mRNAs in context. For a limited number of targets of interest, traditional immunoblotting is widely used to evaluate protein abundance, e.g., [4,6,34,72]. In large-scale investigations of RISC reprogramming, proteomic approaches have been used to interrogate the effects of microRNA overexpression and knockdown on thousands of proteins at once, using stable isotope-labeled cultured cells to provide high-throughput and highly quantitative data [73,74]. Two-dimensional gel electrophoresis has been employed in H9c2 cardiomyocyte-like cells [75] and in mouse hearts overexpressing microRNAs [7,37] but the number of recognizable protein species is relatively small and quantitation is more difficult [76]. One advantage, however, is that microRNA effects on enzyme species such as kinases and phosphatases can be further explored by monitoring phosphoprotein profiles [7].

## 4. Assessing microRNAs not Altered in the Stressed Heart

As shown in Figure 1, while a number of microRNAs are altered during early pressure-overload there are even more that do not change, including some of the most abundant species. In fact, there are likely to be a number of cardiac microRNAs that appear impervious to most stimuli or stressors. Given that mRNAs are expected to be targeted by multiple microRNAs, it is therefore worthwhile to consider which mRNAs they are serving to suppress. Especially in the case of abundant microRNAs, these may potentially buffer mRNAs against fluctuations in other microRNAs or other signals.

CLIP techniques (as discussed in Section 3), if established and validated for the adult heart, would prove invaluable in such determinations, since they are able to provide highly accurate microRNA-mRNA pairing data without the need for a stimulus *vs* control, differential expression experiment. While such techniques are readily applicable to cultured cells, there are significant caveats in transferring information gained from cardiomyocyte-like cells (and perhaps even isolated cardiomyocytes from an adult heart) to the realm of a functioning, intact heart, as will be discussed in Section 5. In the absence of CLIP data, transgenic or knockout microRNA strategies coupled with non-crosslinking RISC immunoprecipitation methods and informatic assistance (as described in Section 3.3.2.) will comprise another source of quantitative microRNA-mRNA interaction data. The presence of mature microRNAs in general is required in the immediate postnatal period, as shown by the early lethality of the cardiac-specific Dicer knockout [77], while miR-133a represents an abundant species that is critically important to the correct embryonic development and postnatal maturation of the heart [5]. However, the aim of experiments designed to elucidate the targets of stably–expressed microRNAs in the adult heart would not be to provide insight into developmental and postnatal growth signaling pathways, but rather what homeostatic functions these microRNAs continue to serve in the adult heart. Knockouts of miRs-208a, -208b and -499 (the ‘myomiR’ family) in the heart have demonstrated an interplay between these microRNAs in specifying myosin expression and myofiber type [31]; knockout of miR-22, one of the most abundant microRNAs in the heart [35], interferes with multiple aspects of cardiac Ca^2+^ control and myofibrillar protein content [8,9].

Interestingly, knockout of miR-378, another highly abundant cardiac species, was without obvious cardiac phenotype under baseline conditions [78] although miR-378 decreases considerably during pressure-overload hypertrophy [79]. It has been noted that microRNA knockout animals may display their most significant phenotypes under conditions of imposed stress [80] when a high number of other transcriptional and translational alterations are taking place and may require effective buffering by microRNAs. It will be of interest to understand which, or all, of the following conditions might be true: the mRNAs most importantly regulated by miR-378 could differ under baseline and stress (hypertrophic) conditions; the mRNAs de-repressed by downregulation of miR-378 may only exhibit a marked biological effect in the context of a hypertrophic stimulus; and significant changes in the regulation of miR-378-targeted mRNAs may only occur when other microRNAs, that co-regulate the same mRNAs, are also affected by a hypertrophic stimulus. Administration of exogenous miR-378 (via adeno-associated viral transduction) ameliorated the degree of hypertrophy and improved cardiac function after pressure overload, which represents a highly desirable outcome [79], but based on our understanding of the pleiotropic nature of microRNAs it is likely that the mechanism underlying the effect involves more than the three MAPK species investigated. A more complete understanding of the functions of stably-expressed (and often highly abundant) microRNAs in the heart may suggest benefits arising from either unleashing, or further repressing, the mRNAs and pathways that they control, in a stressed or diseased state.

## 5. Contextual Interpretation of Direct MicroRNA-mRNA Interaction Data

Consider that the mRNAs under suppression by an individual microRNA will depend on the following factors:

1. mRNA sequence and structural considerations that permit microRNA/RISC-binding. While these parameters are likely to be quite stable among different contexts, what might be the effects of alternate 3′UTRs, other splice variants, or polymorphisms on microRNA binding sites? (A noteworthy example is the human angiotensin II type I receptor polymorphism that disrupts binding of miR-155 [81], a microRNA that also has cardiac functions [82].)
2. Abundances of microRNAs and mRNAs and their possible stoichiometries are likely to differ between cell and tissue contexts.
3. Competition may take place between mRNAs of different abundances but similar binding sites for access to the same microRNA. Furthermore, microRNAs have been reported to bind to long noncoding RNAs (lncRNAs), e.g., [83], amongst other competing endogenous RNAs (ceRNAs) such as circular RNAs [84]; at least one study has cast doubts on the significance of ceRNAs in titrating microRNA availability [85]. Nonetheless, the binding of microRNAs to mRNAs is a process that depends on multiple RNA interaction equilibria and will be altered by abundances of these other participants.

**Figure 4:**
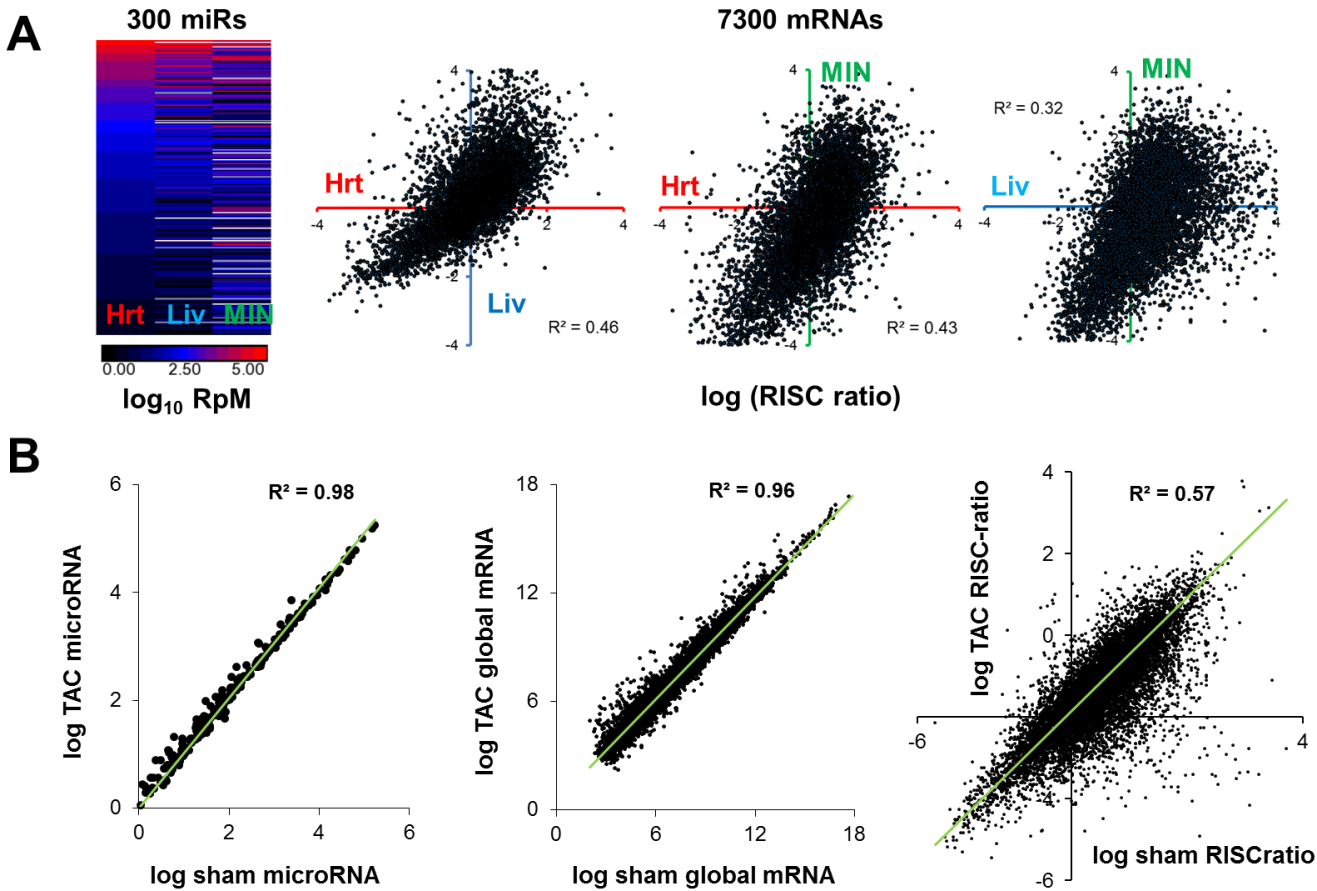
(**a**) *Left*, Heatmap displaying abundances of 300 microRNAs expressed in mouse heart, liver and MIN-6 cultured β-cells. *Right*, graphs of log (RISC ratio), where RISC ratio equals (RISC abundance / global mRNA abundance), for 7300 mRNAs expressed in common between heart (red), liver (blue) and β-cells (green). (**b**) Transcriptome-wide differences in microRNAs, global mRNAs, and mRNA RISC ratios in sham-operated and 1-week pressure-overloaded (TAC) mouse hearts. Data in (**b**) are redrawn from [34] and NCBI GEO accession GSE56890.

The mRNAs under active repression by a microRNA will thus partly depend on cellular context, meaning not just cellular identity (e.g., cardiomyocyte or fibroblast) but also including cellular state (stressed / nonstressed / diseased). Different RNA interaction equilibria that eventually affect the strength of a given microRNA-mRNA association would be expected amongst cells expressing different cohorts of RNAs. An illustration of this point is given in Figure 4a, in which the abundance profiles of a set of microRNAs commonly expressed between mouse heart, liver and cultured islet β-cells are quite dissimilar, and correspondingly the RISC abundance *vs* global abundance ‘ratios’ for a common set of mRNAs are markedly different. Within the same tissue (heart), the contrast of pressure-overload to sham conditions generates considerable differences in the RISC-*vs*-global mRNA ‘ratios’, even though the sum total of the alterations in microRNA and mRNA expression (when averaged over all expressed microRNAs and mRNAs) is relatively slight (Figure 4b).

Armed with a greater understanding of cell-specific microRNA-mRNA interactions, there remains a need to ‘connect the dots’ from the direct mRNA targets of microRNAs, through highly complex webs of interactions and secondary effects, to comprehend the outcome of microRNA changes on the overall transcriptome and proteome. To determine the specific roles and biological importance of individual, microRNA-regulated mRNAs, it will be necessary to ‘stand on the shoulders of giants’; our knowledge of the function of any gene is aided by existing studies on how its encoded protein interacts with others and what signals are thus transduced. While the endeavor is large and constantly benefiting from new data, it is reasonable to suggest that modern knowledge aggregators (e.g., Gene Set Enrichment Analysis from the Broad Institute [86,87], DAVID [88], and MetaCore [89]) will offer insight into how microRNA-regulated mRNAs will make an impact on the downstream responses and interactions represented by global mRNA profiling and ribosome mRNA profiling from hearts. Relatively small changes in individual protein levels are often seen in response to altered microRNAs (e.g. [74]) and it is possible that constitutively-expressed proteins such as cytoskeletal and structural membrane constituents and metabolite processing enzymes, even when taken together, may not represent the most biologically significant targets of microRNAs. Those genes which are highly sensitive to abundance changes; *i.e.*, transcription factors and cofactors, kinases, phosphatases and other post-translational protein modifiers, may be among the most important regulated mRNAs. Our work in early pressure-overload hypertrophy has demonstrated that transcription factors are amongst the most highly microRNA-regulated functional groups [34] and microRNA-targeted transcription factors have been uncovered in studies of cardiac miR-22 overexpression [90] and the ‘myomiR’ family [31]. The work of others has shown microRNA regulation of key kinases [6] and phosphatases [91].

If the direct mRNA targets of a regulated microRNA are involved in well-understood processes (such as sarcoplasmic reticulum Ca^2+^ loading and release, action potential shaping or apoptotic pathways), there may be benefits in delivering exogenous microRNAs or using anti-miRs to turn up or turn down the level of such signals. Conversely, less well-understood gene networks in the heart that are revealed to be regulated by a stress-responsive microRNA may now be judged worthy of deeper characterization. Should the use of a microRNA-based reagent lead to both beneficial and deleterious effects in the stressed heart (hypothetical combinations such as increased contractile force but increased apoptosis, or reduced apoptosis but increased electrical instability), a more complete understanding of the microRNA-affected mRNAs and downstream pathways may permit the application of adjunct therapies so that benefits can be obtained with a minimum of unwanted effects. While the above represent only conjectures, a more complete understanding of the actions of individual stress-responsive cardiac microRNAs and of the way these integrate to affect mRNA translation will likely be necessary to plan future microRNA-based therapeutic interventions.

## 6. Validation, Perturbation and Integration

Before embarking on further mechanistic or even pre-clinical studies, the microRNA-mRNA relationships derived from transcriptome-wide profiling procedures such as those described in the foregoing sections require further validation. One resource for work of this kind is the large number of transgenic animals overexpressing microRNAs of interest in the heart, and a number of germline and conditional microRNA knockout alleles, which suggest the possibility of validating microRNA actions in the appropriate intact heart context. Naturally, the issues at hand with these approaches are familiar, no matter the biomolecule under investigation; namely that transgene-driven expression or knockout strategies may well lead to much greater alterations in individual microRNA levels than those induced during cardiac stress. On the one hand, a ‘larger-than-life’ disruption may be seen as an advantage by facilitating experimental readouts, but on the other hand, limited understanding of the role of a microRNA in a complex system of interactions may mean that some of the resulting effects are other than those predicted. A case in point is that *Myh6*-driven overexpression of miR-378 and miR-499 in the mouse heart, but not of miR-143, leads to secondary changes in a large number of other microRNAs by feedback on their initial transcription [35]. This unexpectedly broad alteration of RISC microRNA programming complicates the assignment of microRNA-dependent mRNA regulation in these hearts to individual microRNAs and adds a further challenge to delineating the precise actions of miR-378 and miR-499 in stressed or unstressed hearts by using transgenic approaches.

Given that attempting to validate the role of a molecule in context often involves perturbing that molecule more than is customary in that context, moving entirely outside the cardiac environment offers an alternative with both advantages and disadvantages. Well-established and widely-used* in vitro* testing platforms include co-transfection of microRNA precursors or mimics with luciferase reporter plasmids harboring putative target cDNA fragments, co-transfection of anti-miRs and luciferase reporters, and co-transfection of ‘target-protection’ oligonucleotides which serve as decoys for microRNA binding sites on target mRNAs (e.g. [72]). In addition, the possibility exists for simultaneous transfection of multiple microRNAs for those mRNAs predicted to be most actively suppressed by a combination of regulated microRNAs. Successful validation assays in these environments demonstrate that particular microRNA-mRNA interactions are biophysically possible, but depending on the differences in expression of other microRNAs, mRNAs, lncRNAs, ceRNAs* etc.* which can compete for binding partners, the correspondence of these* in vitro* data to the magnitude of signaling events involving a particular RNA pair in the heart may be limited. Once again, the challenges brought up by these validation approaches are familiar from mechanistic studies on other biomolecules, and suggest that validation assays need to take multiple forms as a way of overcoming the drawbacks inherent in each.

Despite the imperfections and difficulties involved in judging the correspondence of microRNA-mRNA interaction data obtained outside the heart to the complex situation occurring within, the rewards may be considerable. While biological signaling networks derived with the help of knowledge aggregators [86,87,88,89] may offer much insight into the behavior of complex systems, they tend to be descriptive in nature rather than quantitative, since interaction equilibria and substrate concentrations are highly tissue-dependent. If quantitative effects can be measured or at least closely approximated, circuit design and bioengineering principles may be brought to bear to model network behavior and predict outcomes in a way that a purely descriptive approach cannot. As a result, particular mRNAs may be classified as more important to signaling outcomes than otherwise expected. Two studies using such mathematical modeling but integrating primary experimental findings, involve defining key regulators in the CREB signaling network of neurons [92] and identifying Ras as the primary network ‘hub’ for control of neonatal rat cardiomyocyte hypertrophy [93]. These studies represent examples of the advantages that may be gained by interpreting empirical data using these approaches. While it may be a stretch at present to envisage how such methods could be used to render all the diverse signaling events from initial microRNA-mRNA recognition and suppression, through layers of downstream effects leading to an overall cardiac phenotype, into a predictive network, it seems possible that similar, integrative methods could be used to address the more limited question of how a multiplicity of regulated microRNAs with different affinities for, and accessibility to a given mRNA, affects its eventual translation.

## 7. MicroRNA Therapeutics for Stress Relief?

As stated in the Introduction, much of the current interest in microRNAs in the heart, and in other tissues, is focused on understanding their mechanisms of action sufficiently to contemplate their manipulation for therapeutic purposes. The clinical use of microRNA mimics (including viral expression strategies) and anti-miRs, faces similar hurdles to the use of RNA interference; delivery to the appropriate tissue, effective uptake and intracellular trafficking, and lack of effects on undesired targets. New approaches to delivery and efficacy are under development, spearheaded by the growth of companies invested in microRNA therapeutic approaches (comprehensively reviewed in references [17] and [94]). The unbiased and systematic approaches suggested by this article to the identification of microRNA targets, and the contribution of microRNAs to functional outcomes, should be valuable for optimizing microRNA-based therapies in the heart.

The most popular microRNA ‘prodrug’, widely used in cellular and whole-animal studies, is that of cholesterol-conjugated or locked nucleic acid-modified anti-miRs. While the first of these was developed to target miR-122 in the liver [95] and significant accumulation of anti-miRs in the liver appears unavoidable, such reagents do effectively target microRNAs in the heart even when injected into the peripheral circulation and are stable for relatively long periods. More importantly, certain anti-miRs have salutary effects: an anti-miR-21 reagent inhibits stress-induced cardiac hypertrophy and fibrosis [96] while one directed against miR-208 combats hypertension-induced heart failure in the Dahl salt-sensitive rat [97]. Anti-miRs against miR-34a [98] and the miR-212/132 family [99] offer protection against cardiac pressure-overload while anti-miR-34 reagents also protect against age-dependent cardiac cell death and injury resulting from myocardial infarction [100]; an anti-miR-24 is able to improve the outcome of pressure-overload by inhibiting decompensation after prolonged hypertrophy [101].

**Figure 5:**
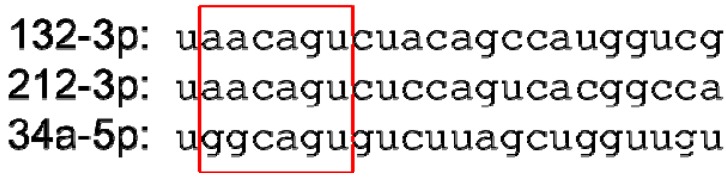
Nucleotide sequences of miRs-132-3p, -212-3p and -34a-5p (following current miRBase [23] nomenclature), given in the 5′ to 3′ direction. A red box denotes the nucleotide 2–7 ‘seed’ sequence.

The fact that all of these reagents have beneficial effects is perhaps sufficient to render precise dissection of their mechanisms of action a moot point. Nonetheless, it is worthwhile to consider that while a cholesterol-conjugated anti-miR-21 antagomiR inhibited stress-induced cardiac hypertrophy and fibrosis [96], treatment with an alternate locked nucleic acid-modified anti-miR-21 or gene ablation of miR-21 had no effect [33]; possible reasons for these disparities have been discussed by both groups [102,103]. While completely speculative, it is interesting that miR-34 and miR-212/132 have a high degree of similarity in the ‘seed’ region (4 contiguous nucleotides, as shown in Figure 5) and given a likely molar excess of anti-miR to endogenous microRNA there is the possibility that these anti-miRs, which have been reported to have similar effects on cardiac hypertrophy [98,99], may utilize at least some mechanisms in common. The foregoing suggests that at least some anti-miR reagents, especially those that target less than the full length of a microRNA, may have effects beyond the initially chosen microRNA. Certainly this does not disqualify them as therapeutics, but it does suggest that caution should be exercised when using such reagents for the dissection or validation of signaling mechanisms in model animals.

## 8. Conclusions

The pursuit of microRNAs as therapeutic vehicles is promising, since it appears they may have higher potential for producing specific biological effects, or at least offer more facile manipulation, than designing strategies for targeting individual enzymes, proteins or other biomolecules. While the likelihood that they will affect multiple mRNAs simultaneously is sometimes quoted as a benefit, it is equally possible that this will prove to be a double-edged sword should beneficial responses be ablated together with deleterious ones. Cells may not give up their regulatory keys as easily as we would like, and their complex circuitry may yield unanticipated effects of manipulating molecules that could represent nodal control points. A rather detailed understanding of microRNA actions might be required to best minimize harmful responses to stress while promoting beneficial ones, through e.g. overriding unwanted anti-miR interference with other signaling modalities via the use of other therapeutics. A great deal of effort from research groups operating at structural, genomic and functional levels has been required to achieve the current level of understanding of microRNAs, and ongoing efforts are needed together with contributions from network biologists and bioengineers to promote our understanding to a higher level. Despite these hurdles, some of the early* in vivo* successes of anti-miRs such as miraversen against hepatitis C [104] and microRNA mimic strategies such as miR-34 against hepatic cancer [105], not to mention the surprising anti-obesity phenotype of anti-miR-208a in mice [106], indicate that delivery methods and chemistry are unlikely to restrict the potential of these molecules; rather, our ability to understand their mechanisms and predict their effects on cells and tissues is likely to prove the limiting factor. With a more comprehensive knowledge of these mechanisms, there is thus hope for exploiting microRNA-regulated signaling networks to optimize the function and survival of hearts responding to the stresses of hypertension, infarct damage, nutrient dysregulation and inflammation.
